# Draft genome sequence of *Flavobacterium* sp. MAHUQ-50 isolated from the rhizosphere of banana in Magura, Bangladesh

**DOI:** 10.1128/mra.00623-25

**Published:** 2025-07-31

**Authors:** Md. Amdadul Huq, Shahina Akter, M. Mizanur Rahman, Md. Anowar Khasru Parvez, Md. Morshedul Alam, Md. Shahedur Rahman

**Affiliations:** 1 Department of Life Sciences, College of BioNano Technology, Gachon University 65440, Seongnam, Republic of Korea; 2 Department of Food Science and Biotechnology, Gachon University 65440, Seongnam, Republic of Korea; 3 Department of Biotechnology and Genetic Engineering, Faculty of Biological Science, Islamic University 247297, Kushtia, Bangladesh; 4 Department of Microbiology, Jahangirnagar University, Dhaka, Bangladesh; 5 Department of Biochemistry and Microbiology, School of Health and Life Sciences, North South University 54495, Dhaka, Bangladesh; 6 Department of Genetic Engineering and Biotechnology, Jashore University of Science and Technology 421984, Jashore, Bangladesh; The University of Arizona, Tucson, Arizona, USA

**Keywords:** genome sequence, *Flavobacterium *sp. MAHUQ-50, rhizosphere

## Abstract

This study reports the draft genome sequence of *Flavobacterium* sp. MAHUQ-50, a strain isolated from the rhizosphere of banana in Magura, Bangladesh, in 2019. The genome consists of a 3,493,675 base pair chromosome assembled into 26 contigs, encoding 3,198 predicted protein coding genes.

## ANNOUNCEMENT

The genus *Flavobacterium* was established by Bergey et al. (1) and emended by Bernardet et al. (2), Kuo et al. (3), Kang et al. (4), and Dong et al. (5). Members of the genus *Flavobacterium* were isolated from different habitats, such as plant, animal, algae, seawater, freshwater, soil, rhizosphere, compost, sediment, lagoon, Antarctic lakes, glacier, activated sludge, water reservoir, and wastewater treatment plant sources (6–8). During investigations on the biodiversity of bacteria in the rhizosphere of banana, a new strain of genus *Flavobacterium*, designated as *Flavobacterium* sp. MAHUQ-50 was isolated. The soil attaching to the plant roots, termed as rhizosphere, is a living place for diverse soil microorganisms. Studying bacterial biodiversity in the plant rhizosphere is crucial because these bacteria play vital roles in plant health, nutrient acquisition, and overall ecosystem functioning (9). Genome-based data have become an essential resource in taxonomic and functional studies of microorganisms. Sequencing bacterial genomes serve as a valuable technique with broad applications in medicine, public health, and evolutionary research. It enables comprehensive analysis of bacteria, identification of biosynthetic gene cluster, detection of virulence-associated genes, monitoring of disease spread, and supports the creation of novel therapeutic agents and vaccines (10–13). This study presents the draft genome assembly of *Flavobacterium* sp. MAHUQ-50.

Strain MAHUQ-50 was isolated from the rhizosphere of banana, located in Dighalgram, Magura, Bangladesh (GPS map: 23.410994, 89.334212). One gram of soil sample was suspended in 9 mL of sterile 0.85% (w/v) NaCl solution. The suspension was serially diluted up to a 10^−6^ dilution, and 100 µL of individual dilution was spread onto R2A agar plates. Then, the plates were placed in a 30°C incubator for 3 days. Single colonies were purified by repeated streaking on fresh R2A agar plates. The genomic DNA was extracted from the bacterial culture that was grown in R2A broth for 24 h using a genomic DNA extraction kit according to the manufacturer’s protocol (Solgent, Republic of Korea). Whole-genome sequencing was performed at the World Data Center for Microorganisms (WDCM), The Institute of Microbiology of the Chinese Academy of Sciences, Beijing 100101, China. The draft genome sequence of the strain MAHUQ-50 was determined using an Illumina HiSeq 3000 platform. The DNA fragments were size selected using AMPure XP beads to isolate those within the desired range, typically 200–300 bp for standard Illumina sequencing libraries. The library for sequencing was prepared using the Nextera XT DNA Library Prep Kit (Illumina, San Diego, USA). After library preparation, sequencing was carried out using the Illumina HiSeq 3000 platform with paired-end 2 × 150 bp reads following the standard protocol provided by the manufacturer. Illumina’s bcl2fastq software version 2.20.0 was utilized with default parameters to demultiplex the raw reads and convert them into FASTQ format. For further downstream analysis, raw paired-end reads from the HiSeq platform were used. Reads were processed with Trimmomatic version 0.38 (14). The genome sequence was assembled by SOAPdenovo v. v2.04; SPAdes v. v3.13.0 assembler (15). The genome was annotated with the NCBI Prokaryotic Genome Annotation Pipeline (PGAP) version 6.3 (16–18). The strain was identified using a whole genome-based taxonomic analysis in the Type (Strain) Genome Server (https://tygs.dsmz.de). Default parameters were used for all software unless otherwise specified. The genome-to-genome distance (GGDH) bioinformatics tool (19) was used to measure *in silico* DNA-DNA hybridization (isDDH) values. The best matches to the strain MAHUQ-50 genome are the type strains *Flavobacterium amniphilum* KYPY10 (accession NZ_JAMLJL000000000) and *Flavobacterium terrae* DSM 18829 (accession GCF_900142035), with *in silico* DNA-DNA hybridization values of 22.3% and 21.0%, respectively.

The genome of *Flavobacterium* sp. MAHUQ-50 is 3,493,675 bp long with a GC content of 35.5%. The assembly contains 26 contigs with a coverage of 248×. A total of 3,258 genes were predicted, of which 3,198 are protein-coding genes. The details of genomic features are presented in Table 1.

**TABLE 1:** Genomic features of *Flavobacterium* sp. MAHUQ-50

Description of source	
Location	Magura, Bangladesh
Time	2019
Type	Rhizosphere of banana
Sequencing summary	
Coverage	248×
Total bases	3,493,675
GC content	35.5%
Assembly report	
Contigs	26
Contig *L*_50_	3
Contig *N*_50_	468.1 kb
Genome length	3.5 Mb
Annotation report	
Genes (total)	3,258
CDSs (total)	3,205
CDSs (with protein)	3,198
ncRNAs	3
tRNA	46
Quality analysis	
Completeness	99.22%
Contamination	0.16%

## Data Availability

The draft genome sequence of Flavobacterium sp. MAHUQ-50 was deposited in GenBank under accession number JBOEFP000000000 . The raw reads are available in SRA under accession number SRR33604969 . The version described in this paper is the first version, JBOEFP000000000.1. The data have been deposited in GenBank under BioSample number SAMN48698540 and BioProject number PRJNA1267049. The genome assembly is available under the accession ASM5063252v1 .
